# Prenatal ultrasound‐based diagnosis of fetal OEIS complex associated with lower limb polymelia and cardiac, hepatic dysplasia: A case report

**DOI:** 10.1002/ccr3.2330

**Published:** 2019-09-28

**Authors:** Jianjun Liu, Yu Liu, Yafang Xue, Yanli Guo

**Affiliations:** ^1^ Department of Ultrasound, Southwest Hospital Army Medical University Chongqing China

**Keywords:** cardiac dysplasia, fetus, hepatic dysplasia, omphalocele‐exstrophy‐imperforate anus‐spinal defects (OEIS) complex, polymelia, prenatal ultrasound diagnosis

## Abstract

OEIS complex is a type of congenital malformation syndrome. Here, we report a case of fetal OEIS complex combined with lower limb polymelia, cardiac defects, and hepatic dysplasia. It was easily misdiagnosed when oligoamnios and the liver bulged. This case will provide reference information for early diagnosis of similar cases.

## Graphical abstract

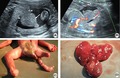

## 1 CASE REPORT

**Figure 1:**
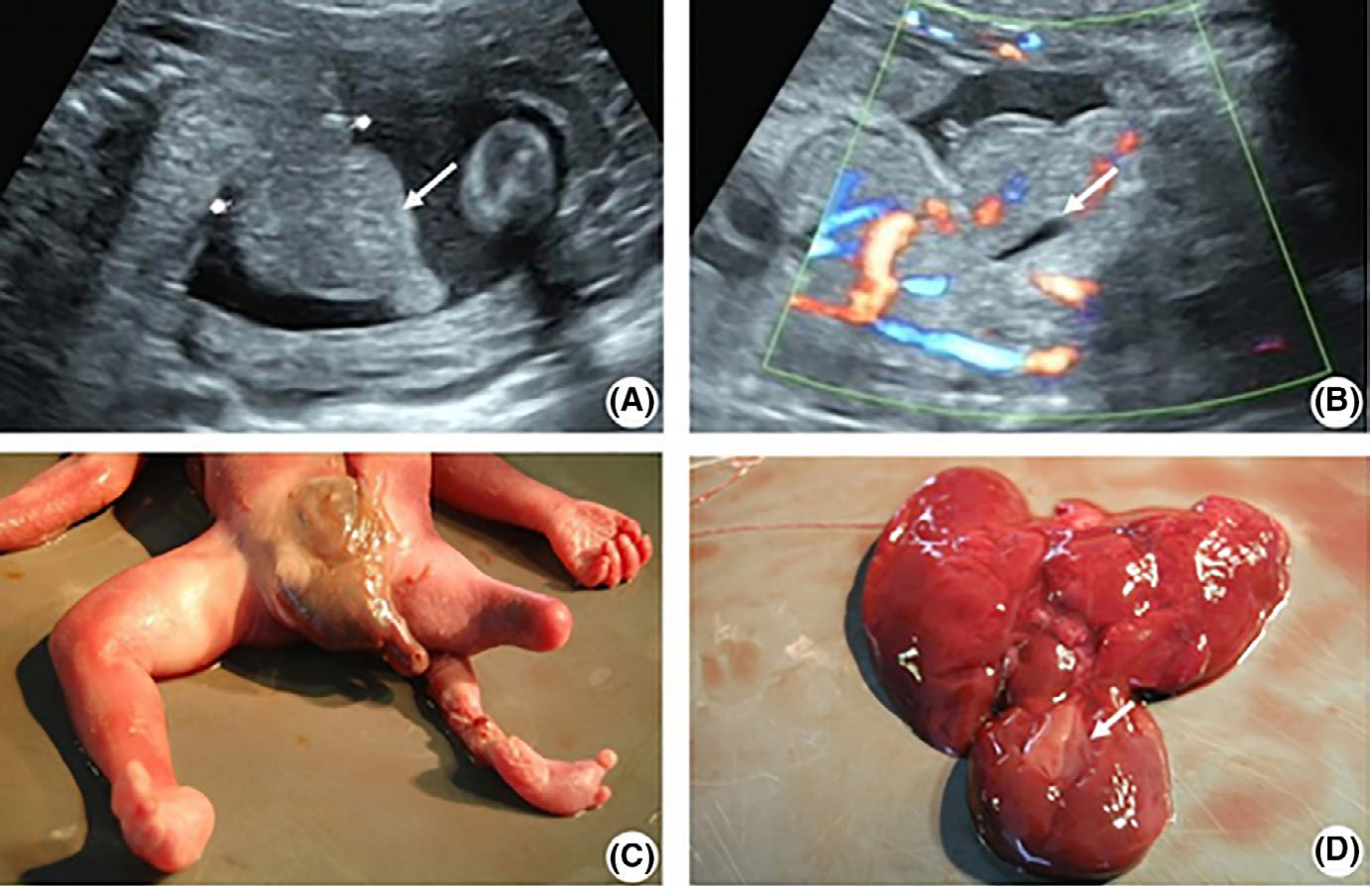
A, Fetal intestine bulge. The white arrow indicates the intestine. B, Part of the liver bulge. The white arrow indicates the fetal gallbladder. C, Photograph of the fetus after induced labor. D, Three‐lobed liver. The white arrow indicates the gallbladder

A 26‐year‐old pregnant woman (gravida 2, para 1), with her first child in good health, presented to our center. Fetal ultrasonography indicated a single live fetus in left breech presentation. The biometric parameters were consistent with the menstrual age at the 24th gestational week. The maximum amniotic fluid pocket was 34 mm. A bidirectional blood flow with an interval of about 1.2 mm was observed at the muscular ventricular septum. In the fetal abdominal image, the bladder, externalia, and the “target loop” of the anus sign were detected as an obscurity. The intestine and a part of the liver bulged to the amniotic cavity on the right side of the anterior wall of the umbilical cord, with the lower edge reaching the perineum (Figure 1A,B). The bulged tissues measured about 35 × 23 mm and were partially capsulated. No kidney was detected in the right renal area, and the adrenal was recumbent. No ectopic kidney was found either in the thoracic or in the abdominal cavities. The medullary cone had moved down. A 9.3 × 5.0 mm echo‐free zone was connected with the spinal cord at the lumbosacral spine region. The left thigh formed a blind end without shank continuation. A long bone was seen at the left thigh root and continued with the foot, which had a fixed posture and only two toes. The right foot presented with a fixed posture and strephenopodia. Furthermore, a single umbilical artery was detected. The ultrasonographic findings indicated a single live fetus with multiple malformations: (a) OEIS complex; (b) absence or ectopia of the right kidney; (c) lower limb polymelia; (d) myelomeningocele; (e) muscular ventricular septal defects; (f) a single umbilical artery; and (g) oligohydramnios. Fetal umbilical cord blood examination by centesis showed that the chromosome karyotype was 46, XX, without chromosomal or karyotypic abnormalities.

Labor was induced at the 25th gestational week. The autopsy showed (a) abnormal development of the abdominal wall: abdominal muscle defect accompanied by bulging of the internal organs; (b) anus atretocystia, that is, vagina opening on the right side of the perineum; (c) lumbosacral myelomeningocele; (d) hepatic dysplasia; (e) abnormal development of the left lower limb; (f) right renal ectopia and dysplasia; (g) muscular ventricular septal defect; and (h) single umbilical artery. The autopsy results confirmed the presence of OEIS complex combined with other multiple deformities including lower limb polymelia, cardiac defects, and hepatic dysplasia, which were consistent with the ultrasonographic findings. The autopsy also confirmed that the left shank with the absence of fibula originated from the thigh root and another femur continued with this shank in the pelvic cavity (Figure 1C). As shown in Figure 1D, the liver was composed of three lobes. The right kidney was ectopic and hypogenetic, located in the right iliac fossa, and with approximately 5 mm of renal parenchyma.

## 2 DISCUSSION

OEIS complex is a type of severe and rare malformation, which can be combined with complex central nervous system malformations, abdominal wall defects, and malformations of the urinary, reproductive, and skeletal systems that lead to death if left untreated.^1^, ^2^ The incidence of OEIS is 0.04%‐0.05% in live births.^3^, ^4^ Differential diagnoses of OEIS include all other forms of abdominal wall defects such as schisis defects, exstrophy of the bladder, and limb‐body wall complex disorders.^5^ In this case, the bulging contents of fetal anterior abdominal wall were located on the lower right side of the umbilical cord, with a relatively low bulge position and large abdominal wall defects. However, the umbilical cord opening was located on the surface of bulging lumps in cases with pure omphalocele. The elephant's trunk‐like intestinal canal at the terminus of the bulge is a characteristic feature of the OEIS complex.^6^ Autopsy results showed intact skin coverage on the spine and downward medullary cone. The myelomeningocele at the lumbosacral portion manifesting as closed spinal bifida is another characteristic feature of the OEIS complex. Therefore, when a low abdominal wall defect with viscus bulging, closed spinal bifida, absent bladder, and ambiguous external genitalia is observed on ultrasound images, the presence of OEIS complex should be considered first. As the cloacal deformity is often combined with multiple malformations, other organs such as the kidneys, limbs, and umbilical artery should also be carefully examined.

In contrast to previous reports, this case of OEIS complex was accompanied by severe lower limb polymelia. Fetal limb polymelia is extremely rare and can easily be misdiagnosed. Moreover, fetal OEIS complex with concurrent limb polymelia and feet dysplasia is rarer. Its exact mechanism remains unclear. Some scientists presume that it is a congenital malformation induced by hereditary genetic defects. The majority consider that limb polymelia is a type of asymmetrical conjoined twin, namely the parasitic fetus. If the fetus encased in the main fetal body is the inner parasitic fetus, the one attached to the surface of main fetal body is the outer parasitic fetus.^7^ The outer parasitic fetus is often attached to the site between the bottom of the main fetal body umbilical cord and the pubis, and usually has stunted truncus and short limbs.^8^, ^9^ In limb polymelia, all nutrition is supplied by the main fetus, and other internal organs may have duplication anomalies or may be directly connected. In this case, the left thigh had a blind end and the left shank and the left foot were absent; moreover, the left shank directly originated at the back of left thigh root. Because of the oligohydramnios, the structures inside the pelvic cavity appeared as obscurity. The autopsy results found the other femur inside the pelvic cavity, which was connected to the shank from the thigh root. Furthermore, the shank fibula was missing and there were only two toes on the connective left foot.

In this case, the fetal OEIS complex was combined with multiple malformations including hepatic dysplasia, renal ectopia, muscular ventricular septal defects, and a single umbilical artery. Therefore, when examining a fetus with OEIS complex, the important organs and structures such as the heart, liver, kidney, limbs, umbilical artery, and vein should be carefully examined to exclude the presence of accompanying malformations. Among these, liver congenital abnormality is rather rare. Abnormalities in liver morphology usually include agenesis, hypoplasia, hypergenesis, and abnormal hepatic fissure. This case developed hypergenesis in the liver. As the omphalocele contains the gallbladder and a part of the liver, liver deformities can be easily missed on ultrasound images. Therefore, even in the state of omphalocele, when examining the liver, the courses of hepatic portal veins and hepatic veins should be carefully observed to systematically analyze the developmental state and presence of congenital abnormalities in the liver.

In summary, we present here a case of OEIS accompanied by polymelia, and our diagnostic experience is as follows: Symmetrical images should be acquired when examining the limbs, which can avoid repeated collection of information on one side of the limb while missing the abnormalities on the other limb. Additionally, both ends of the joints should be included when examining a long bone, and the joint structure and activity of the limbs should be observed.

## CONFLICT OF INTEREST

The authors declare no conflict of interest.

## AUTHOR CONTRIBUTIONS

The author Jianjun Liu is responsible for writing original draft. The author Yu Liu is responsible for review and editing. The author Yafang Xue is responsible for investigation. The author Yanli Guo is responsible for supervision, review, and editing.

## ETHICAL APPROVAL

The pregnant woman provided informed consent or publication of this case. This report follows the principles of the Institutional Review Board (IRB) of Faculty of Medicine, Army Medical University.

## ESTABLISHED FACTS

OEIS complex is a rare and fatal complex deformity. When it is accompanied by other complicated malformations, the ultrasonographic features remain unclear and it is easily misdiagnosed.

## NOVEL INSIGHTS

Besides characteristic ultrasonographic features of OEIS complex, this case was combined with severe and rare lower limb polymelia and multilobe liver deformity. It was easily misdiagnosed when oligoamnios and the liver bulged. This case will strengthen our understanding of such rare deformities and provide reference information for early diagnosis of similar cases.
